# Characterization of perfect numerical semigroups in terms of pseudo-Frobenius numbers

**DOI:** 10.1016/j.heliyon.2024.e33627

**Published:** 2024-07-01

**Authors:** Meng Li, Hui Guo, Ya Tian

**Affiliations:** School of Mathematics and Information Technology, Xingtai University, Xingtai, Hebei, 054001, China

**Keywords:** 05C25, 16U60, Numerical semigroup, Isolated gap, Pseudo-Frobenius

## Abstract

In the theory of numerical semigroups, characterizing numerical semigroups in terms of pseudo-Frobenius numbers is one of the fundamental problems, which is very difficult to achieve in general. This article's main purpose is to answer this problem in the case of perfect numerical semigroups having a Maximal Embedding Dimension (MED).

## 1 Introduction and preliminaries

Let N={1,2,3,…}, and let $N_{0}=N\cup \{0\}$. This paper focuses on the most basic structure known as the numerical semigroup. It is a specific type of semigroup that can be defined as the set

$$S=\langle A\rangle =\{x_{1}a_{1}+\ldots +x_{n}a_{n}:x_{i}\geq 0\},$$

where $A=\{a_{1}<a_{2}<,\ldots ,<a_{n}\}\subseteq N$ with gcd⁡(A)=1. If there does not exist any B⊂A such that S=〈B〉, then A is the set of minimal generators. The positive integers e(S)=#(A) and m(S)= min(A) are Embedding Dimension (ED) e(S) and the Multiplicity (*m*) m(S) of S respectively. The integer e(S) is bounded above by m(S), and if m(S)=e(S) then we say S has a MED. Let N∖S=G(S) be the gap set, then max(G(S))=μ(S) is the Frobenius number of S. The maximal elements of G(S) satisfying the relation, $a_{1}\leq_{S}a_{2}$ if $a_{2}-a_{1}\in S$, are called the set of pseudo-Frobenius numbers denoted by Pμ(S). A gap g∈G(S) is termed an isolated gap if N(g)={g−1,g+1}⊆S. A numerical semigroup is perfect if N(g)⊈S for every g∈G(S). Furthermore, if S is perfect, then μ(S)−1∉S (for more details see [1], [2], [3], [4], [5], [6], [7]).

Numerical semigroups appeared in many other fields of mathematics, like in singularity theory, combinatorics, cryptography, and number theory. The study of S is linked to the problem of finding non-negative integer solutions to the equation $x_{1}a_{1}+x_{2}a_{2}+\ldots +x_{n}a_{n}$, where $\left\{a_{1},a_{2},\ldots ,a_{n}\right\}$ are positive integers. This problem has attracted the attention of many mathematicians, including Frobenius and Sylvester [8], [9], [10]. Interest in numerical semigroups was revitalized in the latter half of the twentieth century because of how they are used in algebraic geometry [11].

The motivation to characterize the S in terms of Pμ(S) comes from the following (see [12] for more details):

Lemma 1 *The numerical semigroup*S*satisfies*

1. S*is symmetric* ⇔ Pμ(S)={μ(S)}*.*
2. S*is pseudo symmetric* ⇔ $P\mu (S)=\{\mu (S),\frac{\mu (S)}{2}\}$*.*

After this, many researchers worked in this direction and gave many interesting results (cf. [13], [14]). The perfect numerical semigroups for the case of e(S)=3, in terms of Pμ(S), are characterized in [3].

Theorem 1.1 *If*e(S)=3*, then*S*is perfect if and only if*Pμ(S)={μ(S),μ(S)−1}*.*

In this article, we focus on characterizing the perfect numerical semigroups of maximal embedding dimensions 4 and 5 in terms of Pμ(S). This characterization is given in Theorem 2.1 and Theorem 3.1. This paper is categorized into four sections. The second and third sections contain parametrization and characterization of perfect numerical semigroups of embedding dimensions 4 and 5. In Section 4, we provide a conclusion that elaborates on our work and also introduces some potential directions for future research.

## 2 Perfect numerical semigroups of MED 4

Here, we characterize the perfect numerical semigroups of MED 4 in terms of Pμ(S).

Lemma 2 *If* $x_{1}\notin S$ *and* $x_{2}\in S$ *then* $x_{1}-x_{2}\notin S$ *.*

Proof If $x_{1}-x_{2}$ belongs to S, then $x_{2}+(x_{1}-x_{2})$ belongs to S. Thus, $x_{1}$ belongs to S.

Lemma 3 *Let* S=〈4,A,B,C〉 *be a numerical semigroup having MED, where* 4<A<B<C *. Then the assertions that follow are true:*

1. *If* 4|(A−1) *and* 4|(B−3) *then* S *is not perfect.*
2. *If* 4|(A−2) *then* S *is not perfect.*
3. *If* 4|(A−3) *and* 4|(B−2) *then* S *is not perfect.*

Proof (1) We may assume that $S=\langle 4,4q_{1}+1,4q_{2}+3,C\rangle$. This implies $q_{1}\leq q_{2}$. If $q_{1}=q_{2}$ then $S=\langle 4,4q_{1}+1,4q_{1}+3,C\rangle$. Clearly, $4q_{1}+2$ is an isolated gap and therefore S is not perfect. Now if $q_{1}<q_{2}$, then let $q_{2}-q_{1}=d>0$. Note that $4q_{2}+1=(4q_{1}+1)+4d\in S$. This implies $4q_{2}+1,4q_{2}+3\in S$. If $4q_{2}+2\in S$ then S is not a maximal embedding dimension, since $C\in \langle 4,4q_{1}+1,4q_{2}+3\rangle$ and if $4q_{2}+2\notin S$ then $4q_{2}+1\in S$ giving $4q_{2}+2$ an isolated gap. Therefore, S cannot be perfect.

(2) We may assume that S=〈4,4q+2,B,C〉. Clearly, 4q,4q+2∈S but 4q+1∉S, i.e., 4q+1 is an isolated gap, and therefore S cannot be perfect.

(3) Since e(S)=4, therefore $S=\langle 4,4q_{1}+3,4q_{2}+2,C\rangle$. This implies $q_{1}<q_{2}$ and $q_{2}-q_{1}=d>0$. Clearly, $4q_{2},4q_{2}+2\in S$. If $4q_{2}+1\in S$ then S is not a maximal embedding dimension, since $C\in \langle 4,4q_{1}+3,4q_{2}+2\rangle$, and if $4q_{2}+1\notin S$, then $4q_{2}+1$ is an isolated gap. Therefore, S cannot be perfect.

In the following proposition, we parameterize the perfect numerical semigroups of MED =4.

Proposition 1 *Let*S*be a perfect numerical semigroup having MED and*e(S)=4*. Then*S*belongs to one of the following two families:*

1. 〈4,x,x+1+4d,x+2+4d〉*, where*x>1*such that*4|(x−1)*and*d≥0*.*
2. 〈4,y,y−2+4d,y−1+4d〉*, where*y>3*such that*4|(y−3)*and*d>0*.*

Proof Assume that S=〈4,A,B,C〉 with 4<A<B<C. Since S is perfect, it follows from Lemma 3 that one of the following two conditions must hold:

1. 4|(A−1), 4|(B−2), and 4|(C−3).
2. 4|(A−3), 4|(B−1), and 4|(C−2).

If condition (1) holds, then $S=\langle 4,4q_{1}+1,4q_{2}+2,4q_{3}+3\rangle$. The possibilities are:

• $q_{1}=q_{2}=q_{3}$,

• $q_{1}=q_{2}<q_{3}$,

• $q_{1}<q_{2}=q_{3}$,

• $q_{1}<q_{2}<q_{3}$.

Consider the case where $q_{3}>q_{2}=q_{1}$. Then $S=\langle 4,4q_{1}+1,4q_{1}+2,4q_{3}+3\rangle$. Clearly, $4q_{1}+1,4q_{1}+2$, and $4q_{1}+4\in S$, but $4q_{1}+3\notin S$. This implies $4q_{1}+3$ is an isolated gap, so S is not perfect.

If $q_{1}<q_{2}<q_{3}$, let $q_{2}-q_{1}=d_{1}$ and $q_{3}-q_{2}=d_{2}$. Since S has MED 4, it follows from [12, Proposition 12] that $\mu (S)=4q_{3}-1$. Note that

$$\mu (S)-1=4q_{3}-2=(4q_{1}+4d_{1}+2)+(d_{2}-1)\cdot 4\in S.$$

This implies S is not perfect. The remaining possibilities are $q_{1}=q_{2}=q_{3}$ or $q_{1}<q_{2}=q_{3}$. Let $x=4q_{1}+1$ and $d=q_{2}-q_{1}\geq 0$, then S has the form 〈4,x,x+1+4d,x+2+4d〉.

If condition (2) holds, then $S=\langle 4,4q_{1}+3,4q_{2}+1,4q_{3}+2\rangle$. The possibilities are:

• $q_{1}<q_{2}=q_{3}$

• $q_{1}<q_{2}<q_{3}$

If $q_{3}>q_{2}>q_{1}$, let $q_{2}-q_{1}=d_{1}$ and $q_{3}-q_{2}=d_{2}$. Then

$$S=\langle 4,4q_{1}+3,4q_{1}+4d_{1}+1,4q_{1}+4d_{1}+4d_{2}+2\rangle .$$

Since S has embedding dimension 4, it follows that $\mu (S)=4q_{3}-2$. Note that

$$\mu (S)-1=4q_{3}-3=(d_{2}-1)\cdot 4+(4q_{2}+1)\in S.$$

This means S is not perfect in this case. The only remaining possibility is $q_{1}<q_{2}=q_{3}$. Let $y=4q_{1}+3$ and $d=q_{2}-q_{1}>0$, then S has the form 〈4,y,y−2+4d,y−1+4d〉.

Theorem 2.1 *Let* S *be a numerical semigroup with MED* e(S)=4 *. Then* S *is perfect if and only if one of the following holds:*

1. Pμ(S)={μ(S)−1,μ(S),μ(S)−(4d+2)} *, where* d≥0 *.*
2. Pμ(S)={μ(S)−1,μ(S),μ(S)−(4d−1)} *, where* d≥1 *.*

Proof (⇒) If S is perfect, then by Proposition 1, S takes one of the following forms:

1. S=〈4,x,x+1+4d,x+2+4d〉, where x>4, 4|(x−1), and d≥0.
2. S=〈4,y,y−2+4d,y−1+4d〉, where y>4, 4|(y−3), and d>0.

First, consider S=〈4,x,x+1+4d,x+2+4d〉. Since S has MED, it follows from [12, Proposition 11 and 12] that

Pμ(S)={x−2+4d,x−3+4d,x−4}.

Here, x−2+4d=μ(S), x−3+4d=μ(S)−1, and x−4=(x−2+4d)−(4d+2)=μ(S)−(4d+2). Thus,

Pμ(S)={μ(S),μ(S)−1,μ(S)−(4d+2)}.

Next, consider S=〈4,y,y−2+4d,y−1+4d〉. Then

Pμ(S)={y−5+4d,y−6+4d,y−4}.

Here, y−5+4d=μ(S), y−6+4d=μ(S)−1, and y−4=(y−5+4d)−(4d−1)=μ(S)−(4d−1). Thus,

Pμ(S)={μ(S),μ(S)−1,μ(S)−(4d−1)}.

(⇐) Assume Pμ(S)={μ(S)−1,μ(S),μ(S)−(4d+2)}. It is easy to see that μ(S)≡3(mod4). Let *g* be an arbitrary gap in S. Then either μ(S)−1−g∈S, μ(S)−g∈S, or μ(S)−(4d+2)−g∈S. If μ(S)−g∈S, then by Lemma 2, μ(S)−1−(μ(S)−g)∉S, i.e., g−1∉S. If μ(S)−1−g∈S, then again from Lemma 2, μ(S)−(μ(S)−1−g)∉S, i.e., g+1∉S. Note that

μ(S)−(4d+2)+1=(μ(S)−1)−4d.

From Lemma 2, it follows that μ(S)−(4d+2)+1∉S. If μ(S)−(4d+2)−g∈S, then Lemma 2 implies that (μ(S)−(4d+2)+1)−(μ(S)−(4d+2)−g)=g+1∉S. Therefore, *g* is not an isolated gap as g−1∉S or g+1∉S.

Now, assume Pμ(S)={μ(S),μ(S)−1,μ(S)−(4d−1)}. Using similar arguments, we can show that if μ(S)−g∈S, then g−1∉S, and if μ(S)−1−g∈S, then g+1∉S. Note that

μ(S)−(4d−1)−1=μ(S)−4d.

From Lemma 2, it follows that μ(S)−(4d−1)−1∉S. Thus, if μ(S)−(4d−1)−g∈S, then by Lemma 2, (μ(S)−(4d−1)−1)−(μ(S)−(4d−1)−g)=g−1∉S. Consequently, S is perfect.

Example 1 Let S=〈4,11,17,18〉 be a numerical semigroup of maximal embedding, and e(S)=4. It is easy to see that the gap set of S is {1,2,3,5,6,7,9,10,13,14} and Pμ(S)={7,13,14}. We show that there is no isolated gap in S. For this, let *g* be an arbitrary gap in S. Then 7−g,13−g or 14−g∈S. Since 6,13,14∉S, we have

6−(7−g)=g−1∉S,

13−(14−g)=g−1∉S,

or

14−(13−g)=g+1∉S.

This means *g* is not an isolated gap, as g−1∉S or g+1∉S. This implies that S is a perfect numerical semigroup.

## 3 Perfect numerical semigroups of MED 5

The following proposition provides us with the conditions on a minimal system of generators for which a numerical semigroup cannot be perfect.

Lemma 4 *Let*S=〈5,a,b,c,d〉*be a numerical semigroup of MED, where*5<a<b<c<d*. Then*

1. *If*5|(a−1)*and*5|(b−3)*, then*S*is not perfect.*
2. *If*5|(a−1)*,*5|(b−4)*, and*5|(c−3)*, then*S*is not perfect.*
3. *If*5|(a−2)*, then*S*is not perfect.*
4. *If*5|(a−3)*and*5|(b−1)*or*5|(b−2)*, then*S*is not perfect.*
5. *If*5|(a−4)*and*5|(b−2)*, then*S*is not perfect.*
6. *If*5|(a−1)*,*5|(b−2)*,*5|(c−4)*, and*5|(d−3)*, then*S*is not perfect.*
7. *If*5|(a−3)*,*5|(b−4)*,*5|(c−2)*, and*5|(d−1)*, then*S*is not perfect.*
8. *If*5|(a−4)*,*5|(b−3)*,*5|(c−2)*, and*5|(d−1)*, then*S*is not perfect.*
9. *If*5|(a−4)*,*5|(b−1)*,*5|(c−3)*, and*5|(d−2)*, then*S*is not perfect.*

Proof (1) Assume $S=\langle 5,5q_{1}+1,5q_{2}+3,c,d\rangle$. This implies $q_{1}\leq q_{2}$. If $q_{1}=q_{2}$, then $5q_{1}+2$ is an isolated gap, making S imperfect. If $q_{1}<q_{2}$, let $q_{2}-q_{1}=d>0$. Note that $5q_{2}+1=(5q_{1}+1)+5d\in S$, implying $5q_{2}+1,5q_{2}+3\in S$. Suppose $5q_{2}+2\in S$. Since *c* and *d* are minimal generators and a≡1(mod5) and b≡3(mod5), either c≡2(mod5) or d≡2(mod5). Assume c≡2(mod5). Then c=5q+2 and $q>q_{2}$, since $c>5q_{2}+2$. Let $t=q-q_{2}$, then $c=(5q_{2}+2)+5t\in \langle 5,5q_{2}+2\rangle$, so *c* is not a minimal generator, a contradiction. Thus, $5q_{2}+2\notin S$ and is an isolated gap.

(2) Assume $S=\langle 5,5q_{1}+1,5q_{2}+4,5q_{3}+3,d\rangle$, giving $q_{1}\leq q_{2}<q_{3}$. Let $q_{2}-q_{1}=d_{1}\geq 0$ and $q_{3}-q_{2}=d_{2}>0$. Note

$$5q_{3}+1=(5q_{1}+1)+5(d_{1}+d_{2})\in S.$$

Suppose $5q_{3}+2\in S$. Since *d* is a minimal generator and d≡2(mod5), d=5q+2 and $q>q_{3}$, as $d>5q_{3}+2$. Let $t=q-q_{3}$, then $d=(5q_{3}+2)+5t\in \langle 5,5q_{3}+2\rangle$, so *d* is not a minimal generator, a contradiction. Thus, $5q_{3}+2\notin S$ and is an isolated gap.

(3) If a≡2(mod5), then 5q,5q+2∈S, but 5q+1∉S, making 5q+1 an isolated gap and S not perfect.

(4) If a≡3(mod5) and b≡1(mod5) or b≡2(mod5), then $S=\langle 5,5q_{1}+3,5q_{2}+1,c,d\rangle$ or $S=\langle 5,5q_{1}+3,5q_{2}+2,c,d\rangle$, both implying $q_{1}<q_{2}$. Clearly, $5q_{1}+4$ is an isolated gap, making S imperfect.

(5) Assume $S=\langle 5,5q_{1}+4,5q_{2}+2,c,d\rangle$, implying $q_{1}<q_{2}$. Clearly, $5q_{2},5q_{2}+2\in S$. Suppose $5q_{2}+1\in S$. Since *c* and *d* are minimal generators and a≡4(mod5) and b≡2(mod5), either c≡1(mod5) and d≡3(mod5) or c≡3(mod5) and d≡1(mod5). Assume c≡1(mod5). Then c=5q+1 and $q>q_{2}$, since $c>5q_{2}+1$. Let $t=q-q_{2}$, then $c=(5q_{2}+1)+5t\in \langle 5,5q_{2}+1\rangle$, so *c* is not a minimal generator, a contradiction. Thus, $5q_{2}+1\notin S$ and is an isolated gap. The other case can be proved similarly.

(6) Assume $S=\langle 5,5q_{1}+1,5q_{2}+2,5q_{3}+4,5q_{4}+3\rangle$. Then we have: $q_{1}=q_{2}=q_{3}<q_{4}$ or $q_{1}=q_{2}<q_{3}<q_{4}$ or $q_{1}<q_{2}=q_{3}<q_{4}$ or $q_{1}<q_{2}<q_{3}<q_{4}$.

If $q_{1}=q_{2}=q_{3}<q_{4}$, then $S=\langle 5,5q_{1}+1,5q_{1}+2,5q_{1}+4,5q_{4}+3\rangle$. Clearly, $5q_{1}+2$ and $5q_{1}+4\in S$, but $5q_{1}+3\notin S$, making $5q_{1}+3$ an isolated gap and S not perfect.

If $q_{1}=q_{2}<q_{3}<q_{4}$, let $q_{3}-q_{1}=d_{1}>0$ and $q_{4}-q_{3}=d_{2}>0$. Since S has MED 5, $\mu (S)=5q_{4}-2$. Note

$$\mu (S)-1=5q_{4}-3=5q_{4}+2-5=(5q_{1}+2)+(d_{1}+d_{2}-1)5\in S.$$

Thus, S is not perfect.

If $q_{1}<q_{2}=q_{3}<q_{4}$ or $q_{1}<q_{2}<q_{3}<q_{4}$, let $q_{2}-q_{1}=d_{1}>0$, $q_{3}-q_{2}=d_{2}\geq 0$, and $q_{4}-q_{3}=d_{3}>0$. Since $\mu (S)-1=5q_{4}-3=5q_{4}+2-5=(5q_{2}+2)+(d_{2}+d_{3}-1)5\in S$, S is not perfect.

(7) Assume $S=\langle 5,5q_{1}+3,5q_{2}+4,5q_{3}+2,5q_{4}+1\rangle$. Then $q_{1}\leq q_{2}<q_{3}<q_{4}$. Let $q_{2}-q_{1}=d_{1}\geq 0$, $q_{3}-q_{2}=d_{2}>0$, and $q_{4}-q_{3}=d_{3}>0$. If $q_{1}<q_{2}$, clearly $5q_{1}+4$ is an isolated gap. If $q_{1}=q_{2}$, then $\mu (S)=5q_{4}-4$. Note

$$\mu (S)-1=5q_{4}-5=5(q_{4}-1)\in S.$$

Thus, S is not perfect.

(8) Assume $S=\langle 5,5q_{1}+4,5q_{2}+3,5q_{3}+2,5q_{4}+1\rangle$. Then $q_{1}<q_{2}<q_{3}<q_{4}$. Let $q_{2}-q_{1}=d_{1}>0$, $q_{3}-q_{2}=d_{2}>0$, and $q_{4}-q_{3}=d_{3}>0$. Since S has MED 5, $\mu (S)=5q_{4}-4$. Note

$$\mu (S)-1=5q_{4}-5=5(q_{4}-1)\in S.$$

Thus, S is not perfect.

(9) Assume $S=\langle 5,5q_{1}+4,5q_{2}+1,5q_{3}+3,5q_{4}+2\rangle$. Then $q_{1}<q_{2}\leq q_{3}<q_{4}$. Let $q_{2}-q_{1}=d_{1}>0$, $q_{3}-q_{2}=d_{2}\geq 0$, and $q_{4}-q_{3}=d_{3}>0$. Since S has MED 5, $\mu (S)=5q_{4}-3$. Note

$$\mu (S)-1=5q_{4}-4=5q_{4}+1-5=(5q_{2}+1)+5(d_{2}+d_{3}-1)\in S.$$

Thus, S is not perfect.

In the following proposition, we parameterize the perfect numerical semigroups of MED 5.

Proposition 2 *Let* S *be a perfect numerical semigroup with MED and* e(S)=5 *. Then* S *falls into one of the following categories:*

1. 〈5,x,x+1,x+2,x+3〉 *, where* x≡1(mod5) *.*
2. $\langle 5,x,x+5d_{1}+1,y,y+1\rangle$ *, where* $x+5d_{1}+1<y$ *,* 5|(x−1) *,* 5|(y−3) *, and* $d_{1}\geq 0$ *.*
3. 〈5,x,y,y+1,y+2〉 *, where* x<y *,* 5|(x−1) *, and* 5|(y−2) *.*
4. 〈5,x,x+1,y,y+1〉 *, where* x+1<y *,* 5|(x−3) *, and* 5|(y−1) *.*
5. $\langle 5,x,x+5d_{1}-3,y,y+1\rangle$ *, where* $x+5d_{1}-3<y$ *,* 5|(x−4) *,* 5|(y−2) *, and* $d_{1}>0$ *.*
6. 〈5,x,y,y+1,y+2〉 *, where* x<y *,* 5|(x−4) *, and* 5|(y−1) *.*
7. $\langle 5,x,x+5d_{1}-1,y,y+1\rangle$ *, where* $x+5d_{1}-1<y$ *,* 5|(x−4) *,* 5|(y−1) *, and* $d_{1}>0$ *.*
8. $\langle 5,x,x+5d_{1}+3,y,y+1\rangle$ *, where* $x+5d_{1}+3<y$ *,* 5|(x−1) *,* 5|(y−2) *, and* $d_{1}>0$ *.*
9. 〈5,x,x+3,y,y+1〉 *, where* x+3<y *,* 5|(x−1) *, and* 5|(y−2) *.*

Proof We can consider S=〈5,a,b,c,d〉 with 5<a<b<c<d. If S is perfect, according to Lemma 4, we have the following scenarios:

• 5|(a−1), 5|(b−2), 5|(c−3), and 5|(d−4).
• 5|(a−3), 5|(b−4), 5|(c−1), and 5|(d−2).
• 5|(a−4), 5|(b−1), 5|(c−2), and 5|(d−3).
• 5|(a−4), 5|(b−3), 5|(c−1), and 5|(d−2).
• 5|(a−1), 5|(b−4), 5|(c−2), and 5|(d−3).

If 5|(a−1), 5|(b−2), 5|(c−3), and 5|(d−4), then $S=\langle 5,5q_{1}+1,5q_{2}+2,5q_{3}+3,5q_{4}+4\rangle$. This implies $q_{1}=q_{2}=q_{3}=q_{4}$ or $q_{1}=q_{2}=q_{3}<q_{4}$ or $q_{1}<q_{2}=q_{3}=q_{4}$ or $q_{1}\leq q_{2}<q_{3}=q_{4}$ or $q_{1}=q_{2}<q_{3}<q_{4}$ or $q_{1}<q_{2}=q_{3}<q_{4}$ or $q_{1}<q_{2}<q_{3}<q_{4}$.

If $q_{1}=q_{2}=q_{3}<q_{4}$, then $S=\langle 5,5q_{1}+1,5q_{1}+2,5q_{1}+3,5q_{4}+4\rangle$. Clearly, $5q_{1}+3,5q_{1}+5\in S$, but $5q_{1}+4\notin S$. This implies $5q_{1}+4$ is an isolated gap. Thus, S is not perfect.

If $q_{1}=q_{2}<q_{3}<q_{4}$, then let $q_{3}-q_{1}=d_{1}>0,q_{4}-q_{3}=d_{2}>0$. Since S is a numerical semigroup with MED 5, therefore $\mu (S)=5q_{4}-1$. Note that

$$\mu (S)-1=5q_{4}-2=5q_{4}+3-5=(5q_{3}+3)+(d_{2}-1)5\in S.$$

This implies that S is not perfect.

If $q_{1}<q_{2}\leq q_{3}<q_{4}$, then let $q_{2}-q_{1}=d_{1}>0,q_{3}-q_{2}=d_{2}\geq 0$, and $q_{4}-q_{3}=d_{3}>0$. Since S is a numerical semigroup with MED 5, therefore $\mu (S)=5q_{4}-1$. Note that

$$\mu (S)-1=5q_{4}-2=5q_{4}+3-5=(5q_{3}+3)+(d_{3}-1)5\in S.$$

This implies that S is not perfect.

If $q_{1}=q_{2}=q_{3}=q_{4}$ then $S=\langle 5,5q_{1}+1,5q_{1}+2,5q_{1}+3,5q_{1}+4\rangle$. Consider $x=5q_{1}+1$, then we get (1). Now if $q_{1}\leq q_{2}<q_{3}=q_{4}$, then $S=\langle 5,5q_{1}+1,5q_{1}+5d_{1}+2,5q_{3}+3,5q_{3}+4\rangle$, where $q_{2}-q_{1}=d_{1}\geq 0$. Consider $x=5q_{1}+1$ and $y=5q_{3}+3$. This gives (2). Also, if $q_{1}<q_{2}=q_{3}=q_{4}$, then $S=\langle 5,5q_{1}+1,5q_{2}+2,5q_{2}+3,5q_{2}+4\rangle$. Assume $x=5q_{1}+1$ and $y=5q_{2}+2$, then we get (3). A Similar discussion of the remaining cases gives the parametrizations (4) to (9).

In the subsequent Theorem, we provide a characterization of perfect numerical semigroups with MED 5 in terms of pseudo-Frobenius numbers.

Theorem 3.1 *Let*S*be a numerical semigroup with maximal embedding, and*e(S)=5*. Then*S*is perfect if and only if one of the following conditions holds:*

1. $P\mu (S)=\{\mu (S)>\mu (S)-1>\mu (S)-2>\mu (S)-(5d_{1}+3)\}$*, where*$0\leq d_{1}$*.*
2. $P\mu (S)=\{\mu (S)>\mu (S)-1>\mu (S)-(5d_{2}+2)+5d_{1}>\mu (S)-(5d_{2}+3)\}$*, where*$0\leq d_{1}\leq d_{2}$*.*
3. $P\mu (S)=\{\mu (S)>\mu (S)-1>\mu (S)-(5d_{1}-2)>\mu (S)-(5d_{1}-1)\}$*, where*$1\leq d_{1}$*.*
4. $P\mu (S)=\{\mu (S)>\mu (S)-1>\mu (S)-(5d_{2}+2)+5d_{1}>\mu (S)-(5d_{2}-1)\}$*, where*$1\leq d_{1}\leq d_{2}$*.*
5. $P\mu (S)=\{\mu (S)>\mu (S)-1>\mu (S)-2>\mu (S)-(5d_{1}-1)\}$*, where*$1\leq d_{1}$*.*
6. $P\mu (S)=\{\mu (S)>\mu (S)-1>\mu (S)-(5d_{2}-1)+5d_{1}>\mu (S)-(5d_{2}-2)\}$*, where*$1\leq d_{1}<d_{2}$*.*
7. $P\mu (S)=\{\mu (S)>\mu (S)-1>\mu (S)-(5d_{2}-1)+5d_{1}>\mu (S)-(5d_{2}+2)\}$*, where*$0\leq d_{1}<d_{2}$*.*
8. $P\mu (S)=\{\mu (S)>\mu (S)-1>\mu (S)-(5d_{1}-1)>\mu (S)-(5d_{1}+2)\}$*, where*$1\leq d_{1}$*.*

Proof (⇒) Assume S=〈5,a,b,c,d〉 with 5<a<b<c<d. If S is perfect, then it must conform to one of the forms (1) to (9) outlined in Proposition 2.

Let S=〈5,x,x+1,x+2,x+3〉. Since S has MED,

Pμ(S)={x−2,x−3,x−4,x−5},withμ(S)=x−2.

This implies

Pμ(S)={μ(S),μ(S)−1,μ(S)−2,μ(S)−3}.

If S=〈5,x,y,y+1,y+2〉, then

Pμ(S)={y−3,y−4,y−5,x−5},withμ(S)=y−3.

Given x≡1mod5 and y≡2mod5, we set $x=5q_{1}+1$ and $y=5q_{2}+2=5q_{1}+5d_{1}+2$ with $d_{1}=q_{2}-q_{1}$. Note that $x-5=(5q_{1}+5d_{1}+2-3)-(5d_{1}+3)=F-(5d_{1}+3)$. This yields

$$P\mu (S)=\{\mu (S),\mu (S)-1,\mu (S)-2,\mu (S)-(5d_{1}+3)\},\;\text{where}\;\;d_{1}>0.$$

If $S=\langle 5,x,x+5d_{1}+1,y,y+1\rangle$, then

$$P\mu (S)=\{y-4,y-5,x+5d_{1}-4,x-5\},\;\text{with}\;\;\mu (S)=y-4.$$

Given x≡1mod5 and y≡3mod5, we set $x=5q_{1}+1$ and $y=5q_{2}+3=5q_{1}+5d_{2}+3$, where $d_{2}=q_{2}-q_{1}$. Note that

$$x-5=(5q_{1}+5d_{2}+3-4)-(5d_{2}+3)=\mu (S)-(5d_{2}+3),$$

$$x+5d_{1}-4=(5q_{1}+5d_{2}+3-4)-(5d_{2}+2)+5d_{1}=\mu (S)-(5d_{2}+2)+5d_{1}.$$

This gives

$$P\mu (S)=\{\mu (S),\mu (S)-1,\mu (S)-(5d_{2}+2)+5d_{1},\mu (S)-(5d_{2}+3)\}.$$

Remaining cases can be handled similarly.

(⇐) Let $P\mu (S)=\{\mu (S),\mu (S)-1,\mu (S)-2,\mu (S)-(5d_{1}+3)\}$ and *g* be a gap in S. Then μ(S)−g∈S,μ(S)−1−g∈S,μ(S)−2−g∈S, or $\mu (S)-(5d_{1}+3)-g\in S$. If μ(S)−g∈S, then Lemma 2 implies μ(S)−1−(μ(S)−g)∉S, i.e., g−1∉S. If μ(S)−1−g∈S, then from Lemma 2, we have μ(S)−(μ(S)−1−g)∉S, i.e., g+1∉S. If μ(S)−2−g∈S, then again Lemma 2 implies μ(S)−1−(μ(S)−2−g)∉S, i.e., g+1∉S. Now if $\mu (S)-(5d_{1}+3)-g\in S$, then note that $\mu (S)-(5d_{1}+3)+1=5q_{1}-3\notin S$. Then, from Lemma 2, we have $\mu (S)-(5d_{1}+3)+1-(\mu (S)-(5d_{1}+3)-g)\notin S$, i.e., g+1∉S. This implies *g* is not an isolated gap as g−1∉S or g+1∉S.

Now if $P\mu (S)=\{\mu (S),\mu (S)-1,\mu (S)-(5d_{2}+2)+5d_{1},\mu (S)-(5d_{2}+3)\}$, then employing analogous arguments as above, we can demonstrate that μ(S)−g∈S yields g−1∉S and μ(S)−1−g∈S implies g+1∉S. Note that

$$\mu (S)-(5d_{2}+2)+5d_{1}+1=(\mu (S)-1)-(d_{2}-d_{1})5.$$

Given $d_{2}-d_{1}\geq 0$, Lemma 2 entails $\mu (S)-(5d_{2}+2)+5d_{1}+1\notin S$. If $\mu (S)-(5d_{2}+2)+5d_{1}-g\in S$, then again Lemma 2 implies $(\mu (S)-(5d_{2}+2)+5d_{1}+1)-(\mu (S)-(5d_{2}+2)+5d_{1}-g)\notin S$, i.e., g+1∉S.

If $\mu (S)-(5d_{2}+3)-g\in S$, then

$$\mu (S)-(5d_{2}+3)+1=(\mu (S)-(5d_{2}+2)+5d_{1})-5d_{1}\notin S.$$

This implies $\mu (S)-(5d_{2}+3)+1-(\mu (S)-(5d_{2}+3)-g)\notin S$, i.e., g+1∉S. This implies *g* is not an isolated gap as g−1∉S or g+1∉S.

If $P\mu (S)=\{\mu (S),\mu (S)-1,\mu (S)-(5d_{1}-2),\mu (S)-(5d_{1}-1)\}$, then $\mu (S)-(5d_{1}-2)-1=\mu (S)-(5d_{1}-1)\notin S$. Now, suppose $\mu (S)-(5d_{1}-2)-g\in S$. Then $(\mu (S)-(5d_{1}-2)-1)-(\mu (S)-(5d_{1}-2)-g)\notin S$, i.e., g−1∉S. Similarly, if $\mu (S)-(5d_{1}-1)-g\in S$, then $\mu (S)-(5d_{1}-1)+1=\mu (S)-(5d_{1}-2)\notin S$, which implies $\mu (S)-(5d_{1}-1)+1-(\mu (S)-(5d_{1}-1)-g)\notin S$, i.e., g+1∉S. Therefore, *g* is not an isolated gap as g−1∉S or g+1∉S.

Now, if $P\mu (S)=\{\mu (S),\mu (S)-1,\mu (S)-(5d_{2}+2)+5d_{1},\mu (S)-(5d_{2}-1)\}$, then $\mu (S)-(5d_{2}+2)+5d_{1}+1=(\mu (S)-1)-(d_{2}-d_{1})5\notin S$. So, if $\mu (S)-(5d_{2}+2)+5d_{1}-g\in S$, then $(\mu (S)-(5d_{2}+2)+5d_{1}+1)-(\mu (S)-(5d_{2}+2)+5d_{1}-g)\notin S$, i.e., g+1∉S. Similarly, if $\mu (S)-(5d_{2}-1)-g\in S$, then $\mu (S)-(5d_{2}-1)-1=5q_{1}-2\notin S$. This implies $\mu (S)-(5d_{2}-1)-1-(\mu (S)-(5d_{2}-1)-g)\notin S$, i.e., g−1∉S. Hence, *g* is not an isolated gap as g−1∉S or g+1∉S.

Let $P\mu (S)=\{\mu (S),\mu (S)-1,\mu (S)-2,\mu (S)-(5d_{1}-1)\}$, then $\mu (S)-(5d_{1}-1)-1=\mu (S)-5d_{1}\notin S$. This implies if $\mu (S)-(5d_{1}-1)-g\in S$, then $\mu (S)-(5d_{1}-1)-1-(\mu (S)-(5d_{1}-1)-g)\notin S$, i.e., g−1∉S. Hence, *g* is not an isolated gap as g−1∉S or g+1∉S.

If $P\mu (S)=\{\mu (S),\mu (S)-1,\mu (S)-(5d_{2}-1)+5d_{1},\mu (S)-(5d_{2}-2)\}$, then $\mu (S)-(5d_{2}-1)+5d_{1}-1=\mu (S)-(d_{2}-d_{1})5\notin S$. Now, if $\mu (S)-(5d_{2}-1)+5d_{1}-g\in S$, then $(\mu (S)-(5d_{2}-1)+5d_{1}-1)-(\mu (S)-(5d_{2}-1)+5d_{1}-g)\notin S$, i.e., g−1∉S. Similarly,

$$\mu (S)-(5d_{2}-2)-1=(\mu (S)-(5d_{2}-1)+5d_{1})-5d_{1}\notin S.$$

This implies that if $\mu (S)-(5d_{2}-2)-g\in S$, then $\mu (S)-(5d_{2}-2)-1-(\mu (S)-(5d_{2}-2)-g)\notin S$, i.e., g−1∉S. Therefore, *g* is not an isolated gap as g−1∉S or g+1∉S.

Now, if $P\mu (S)=\{\mu (S),\mu (S)-1,\mu (S)-(5d_{2}-1)+5d_{1},\mu (S)-(5d_{2}+2)\}$, then $\mu (S)-(5d_{2}-1)+5d_{1}-1=\mu (S)-(d_{2}-d_{1})5\notin S$. Hence, if $\mu (S)-(5d_{2}-1)+5d_{1}-g\in S$, then $(\mu (S)-(5d_{2}-1)+5d_{1}-1)-(\mu (S)-(5d_{2}-1)+5d_{1}-g)\notin S$, i.e., g−1∉S.

Also, note that $\mu (S)-(5d_{2}+2)+1=(\mu (S)-1)-5d_{2}\notin S$. Therefore, if $\mu (S)-(5d_{2}+2)-g\in S$ and $\mu (S)-(5d_{2}+2)+1-(\mu (S)-(5d_{2}+2)-g)\notin S$, i.e., g+1∉S, then *g* is not an isolated gap as g−1∉S or g+1∉S.

Now, if $P\mu (S)=\{\mu (S),\mu (S)-1,\mu (S)-(5d_{1}-1),\mu (S)-(5d_{1}+2)\}$, then $\mu (S)-(5d_{1}-1)-1=\mu (S)-5d_{1}\notin S$. Therefore, if $\mu (S)-(5d_{1}-1)-g\in S$, then $(\mu (S)-(5d_{1}-1)-1)-(\mu (S)-(5d_{1}-1)-g)\notin S$, i.e., g−1∉S. Similarly, note that $\mu (S)-(5d_{1}+2)+1=(\mu (S)-1)-5d_{1}\notin S$. Therefore, if $\mu (S)-(5d_{1}+2)-g\in S$ and $\mu (S)-(5d_{1}+2)+1-(\mu (S)-(5d_{1}+2)-g)\notin S$, i.e., g+1∉S, then *g* is not an isolated gap as g−1∉S or g+1∉S.

As a result, S is perfect.

Example 2 Let S=〈5,11,17,23,24〉 be a numerical semigroup of MED with e(S)=5. It is evident that the gap set of S is {1,2,3,4,6,7,8,9,12,13,14,18,19} and Pμ(S)={6,12,18,19}. Now, we demonstrate that there are no isolated gaps in S. Let *g* be an arbitrary gap in S. Then, either 6−g, 12−g, 18−g, or 19−g∈S. Since 7,13,18, and 19 are not elements of S, we deduce that

7−(6−g)=g+1∉S,

13−(12−g)=g+1∉S,

19−(18−g)=g+1∉S,

or

18−(19−g)=g−1∉S.

Thus, *g* is not an isolated gap, as g−1∉S or g+1∉S. This implies that S is a perfect numerical semigroup.

## 4 Conclusion

Firstly, we established the conditions on the generating set under which the numerical semigroup achieves perfection. Employing these conditions, we parameterized the perfect numerical semigroups. Finally, we delineated perfect numerical semigroups in relation to pseudo-Frobenius numbers.

**Future work:** It remains to characterize perfect numerical semigroups with maximal embedding dimensions exceeding 5.

## CRediT authorship contribution statement

**Meng Li:** Writing – review & editing, Validation, Methodology, Investigation, Formal analysis, Conceptualization. **Hui Guo:** Writing – original draft, Validation, Methodology, Investigation, Formal analysis, Conceptualization. **Ya Tian:** Writing – original draft, Validation, Methodology, Investigation, Formal analysis, Conceptualization.

## Declaration of Competing Interest

The authors declare that they have no known competing financial interests or personal relationships that could have appeared to influence the work reported in this paper.

## Data Availability

No data was used for the research described in the article.

## References

[1] Smith H.J. (2022). On isolated gaps in numerical semigroups. Turk. J. Math..

[2] Moreno-Frías M.Á., Rosales J.C. (2019). Perfect numerical semigroups. Turk. J. Math..

[3] Moreno-Frías M.Á., Rosales J.C. (2020). Perfect numerical semigroups with embedding dimension three. Publ. Math. (Debr.).

[4] Xu P., Binyamin M.A., Aslam A., Ali W., Mahmood H., Zhou H. (2020). Characterization of graphs associated with the ideal of numerical semigroups. J. Math..

[5] Fröberg R., Gottlieb C., Häggkvist R. (1987). On numerical semigroups. Semigroup Forum.

[6] Barucci V., Dobbs D.E., Fontana M. (1997).

[7] Wang Y., Binyamin M.A., Ali W., Aslam Adnan, Rao Yongsheng (2021). Graphs associated with the ideals of a numerical semigroup having metric dimension 2. Math. Probl. Eng..

[8] Selmer E.S. (1977). On the linear Diophantine problem of Frobenius. J. Reine Angew. Math..

[9] Sylvester J.J. (1884). Mathematical questions with their solutions. Educ. Times.

[10] Rödseth Ö.J. (1978). On a linear Diophantine problem of Frobenius. J. Reine Angew. Math..

[11] Høholdt T., Van Lint J.H., Pellikaan R. (1998). The Handbook of Coding Theory, vol. 1.

[12] Assi A., García-Sánchez P.A. (2016).

[13] Delgado M., García-Sánchez P.A., Robles-Pérez A.M. (2016). Numerical semigroups with a given set of pseudo-Frobenius numbers. LMS J. Comput. Math..

[14] Robles-Pérez A.M., Rosales J.C. (2016). The genus, Frobenius number and pseudo-Frobenius numbers of numerical semigroups of type 2. Proc. R. Soc. Edinb., Sect. A, Math..

